# Maternal prenatal paracetamol ingestion and scholastic attainments of the offspring

**DOI:** 10.3389/fphar.2023.1116683

**Published:** 2023-12-12

**Authors:** Jean Golding, Holly Tunstall, Steven Gregory, Yasmin lies-Gaven

**Affiliations:** Population Health Sciences, Bristol Medical School, University of Bristol, Bristol, United Kingdom

**Keywords:** ALSPAC, paracetamol, pregnancy, child outcomes, reading, mathematics, spelling, educational ability

## Abstract

**Background:** Fetal exposure to paracetamol (acetaminophen) has been shown to be associated with asthma and other atopic disorders, as well as behavioural problems including hyperactivity, in childhood. However, there is little information on scholastic abilities among children exposed to paracetamol in pregnancy.

**Objectives:** To determine whether there are any differences in scholastic abilities among the offspring of women who ingested paracetamol during pregnancy compared with non-exposed children.

**Methods:** Mothers enrolled in the Avon Longitudinal Study of Parents and Children (ALSPAC) had recorded the frequency with which they had taken the medication over two time periods during pregnancy: i) the first 18 weeks and ii) 18–32 weeks. The offspring have been followed up ever since. For this study we use as outcomes: a) 14 tests of ability at reading and 2 of spelling using the study’s tests and the national education system test results; b) 6 of mathematical abilities including tests of arithmetic and mathematical reasoning, and c) 1 of scientific understanding. Multiple regression was used, adjusting for 15 different exposures including reasons for taking the medication as well as demographic features.

**Results:** Almost all unadjusted and adjusted mean differences were negative (i.e., those exposed to maternal intake of paracetamol did less well), but negative associations for exposures between 18 and 32 weeks of gestation were much more evident than for exposures earlier in pregnancy. Of the later exposures, after adjustment, 12 of the 23 scholastic tests were associated with prenatal exposure to paracetamol at *p* < 0.05. These negative effects were found in the girls (12 tests at *p* < 0.05) but not boys (0 tests at *p* < 0.05).

**Conclusion:** Evidence from this longitudinal study suggests that maternal exposure to paracetamol is associated with disadvantages to the offspring in scholastic abilities such as mathematics and reading at secondary school ages. This raises the question as to whether there are longer-lasting effects on educational attainment from age 15 years onwards, including at university level. Clearly these results should be tested in other settings, but meanwhile they add to a growing accumulation of known adverse effects of exposure to paracetamol in pregnancy.

## Introduction

Paracetamol (*N*-acetyl-*p*-aminophenol, otherwise known as acetaminophen) is the medication most frequently taken by pregnant women. For decades it was assumed to be safe, but more recently several reviews of observational studies have indicated that there are causes for concern (Thiele et al., 2013; Liew and Ernst, 2021; Brune et al., 2015; Bührer et al., 2021; Patel et al., 2022]. These centre around asthma and other atopic disorders; behaviour disorders, particularly attention deficit hyperactivity disorder (ADHD); and disorders of sexual development. Most studies have focussed on early childhood and little attention has been paid to later development, including to scholastic abilities.

Here we use data collected by the Avon Longitudinal Study of Parents and Children (ALSPAC), a British pre-birth cohort which collected information prospectively through pregnancy. The offspring have been followed up since their births in 1991–1992. Previous studies using these data have shown associations between maternal paracetamol intake and wheezing and asthma (Shaheen et al., 2002; 2007] and neurobehavioural features including hyperactivity (Golding et al., 2020) and oppositional-defiant disorder (Ruisch et al., 2018]. However, only a small number of studies of prenatal exposure on cognitive function has been carried out in ALSPAC or other birth cohorts: a small number of studies of IQ levels of the offspring mainly showed no effect (Streissguth et al., 1987; Laue et al., 2019) although one found a lower IQ level among 5 year old children of mothers who had taken paracetamol in the absence of fever, but no association when the mother had taken the medication when feverish (Liew et al., 2016]. In ALSPAC only weak negative associations were found between paracetamol taken by the mother between 18- and 32-weeks gestation and IQ at age eight (Golding et al., 2020). We could find no studies assessing whether offspring who had been exposed to paracetamol *in utero* were achieving at levels expected on school subjects such as reading and mathematics. This, then, is probably the first study to address the question of scholastic abilities at primary and early secondary school ages among children whose mothers had taken paracetamol during pregnancy. Our main hypothesis is that there are adverse effects on the scholastic ability of the child but concede that it may be that the sexes may respond differently from one another in this regard.

## Material and methods

### The ALSPAC population

ALSPAC was designed to determine ways in which different aspects of the environment influence the health and development of children. Pregnant women resident in the Avon area of southwest England were eligible if their expected date of delivery was between 1st April 1991 and 31st December 1992 (Golding et al., 2001; Boyd et al., 2013; Fraser et al., 2013). The initial number of pregnancies enrolled was 14,541 (for these at least one questionnaire has been returned or a “Children in Focus” clinic had been attended by 19th July 1999). Of these initial pregnancies, there was a total of 14,676 foetuses, resulting in 14,062 live births and 13,988 children who were alive at 1 year of age.

Data were collected throughout pregnancy and childhood with self-completion questionnaires to the mothers and later to the offspring themselves. In parallel the children were examined and given tests in clinics especially held by the study, schools carried out tests designed for the study and the results of national tests of scholastic ability were linked to the data base. The study website contains details of all the data that are available through a fully searchable data dictionary and variable search tool: http://www.bristol.ac.uk/alspac/researchers/our-data/

Of those mothers who enrolled in pregnancy, the proportion who responded at various time-points varied with the type of information collected (self-completion questionnaires having higher response rates than hands-on examinations, where the mother and child had to attend a clinic for assessment). The major reason for non-response was a loss to follow-up (for questionnaires) and a household move far from the clinic (for hands-on assessments). Linkage to national educational test results was carried out independently, and was not biased by social circumstances, but omitted children who were resident outside of England. Thus, the response rates varied for the different instruments used. Details can be obtained from the ALSPAC website (www.bristol.ac.uk/alspac/researchers/our-data/).

At the time when the study was being planned and piloted (1988-9) there were no appropriate independent ethics committees in the University of Bristol. It was therefore decided to set up an independent committee to oversee the study (the ALSPAC Ethics and Law Committee. (ALEC)). This was particularly important since the study design was innovative in a number of respects and planning involved substantive data collection. At one stage during the early months, the Committee was meeting several times a month. ALEC has remained independent of ALSPAC throughout and has been approved by the American Institutional Review Board (IRB no.00003312). Subsequently ethical approval for the study was obtained at frequent intervals from this and the Local National Health Service Research Ethics Committees when appropriate. Detailed information on the ways in which confidentiality of the cohort is maintained may be found on the study website: http://www.bristol.ac.uk/alspac/researchers/research-ethics/

Implied consent for the use of data collected via questionnaires was obtained from participants following the recommendations of ALEC at the time (see Birmingham (2018) for further details).

At age 18, study children were sent ‘fair processing’ materials describing ALSPAC’s intended use of their health and administrative (including education) records and were given clear means to consent or object via a written form. Data were not extracted for participants who objected, or who were not sent fair processing materials.

### The exposure

The questions asked in pregnancy were administered in structured self-completion questionnaires at 18 and 32 weeks. The wording at 18 weeks was:

*‘*Please indicate how often you have taken the following pills during this pregnancy’, and at 32 weeks it was: ‘Please indicate how often you have taken the following pills in the last 3 months’. The medications listed at each time point were: a) Aspirin; b) Paracetamol; c) Codeine; d) Mogodon or other sleeping tablets; e) Valium or other tranquilliser. Possible responses were: Everyday; Most days; Sometimes; Not at all. In this paper we concentrate on paracetamol alone. The frequency with which the women reported taking paracetamol at the two time periods is shown in Table 1. In all, 9,207 women answered the question at both time points, 41% of whom had taken paracetamol at both time points.

**TABLE 1:** Frequency with which paracetamol was taken during pregnancy.

Time period	Every day (%)	Most days (%)	Sometimes (%)	Not at all (%)	N^a^
Prior to 18 weeks	0.2	1.4	53.0	45.5	13,147
18–32 weeks	0.2	0.9	42.8	56.1	12,025

^a^ Number answering the question.

#### The outcomes

A total of 23 different measures were used as shown in Table 2 and described in detail in the Supplementary Material. Included were 14 measures related to reading (including speed and accuracy, real words and non-words, comprehension and phoneme awareness) administered at ages ranging from 7 to 13; two spelling tests at ages 7 and 9; six mathematics tests including mental arithmetic and mathematical reasoning at ages from 8 to 13, and one test of scientific reasoning at age 11–12 years.

**TABLE 2:** Descriptors of all scholastic measures used.

Variable	Mean	SD	Range
Reading score at 7	28.09	9.43	0–52
Reading score at 9	7.50	2.50	0–10
Comprehension score at 9	24.95	7.85	0–44
Reading speed at 9	80.66	27.71	14–39
Reading accuracy at 9	66.01	20.56	0–100
Reading speed at 12	77.37	10.59	19–104
Reading speed at 13	82.53	10.34	18–104
Phoneme score at 7	19.99	9.56	0–40
Non-word reading at 9	5.20	2.50	0–10
Non-word reading at 12	49.09	9.62	2–63
Non-word reading at 13	50.80	9.41	4–63
SATs reading assessment at 10-11	4.99	0.89	2–7
KS3 reading test at 13-14	15.11	6.49	0–32
KS3 Shakespeare reading at 13-14	6.71	3.52	0–18
Spelling score at 7	7.73	4.44	0–15
Spelling score at 9	10.19	3.49	0–15
WISC arithmetic score at 8	14.77	3.45	0–28
Year 4 maths test score at 8	10.46	3.16	0–17
Year 6 maths test score at 11	18.78	7.09	0–35
Year 8 maths test score at 12	22.85	7.35	0–35
KS2 maths test score at 10-11	62.57	21.66	0–100
KS3 maths test score at 13-14	80.00	22.32	1–148
Science reasoning score at 11-12	5.75	2.59	0–10

KS2 = Key stage 2; KS3 = Key stage 3 (see Supplementary material); SATs, Standardised Attainment Test Scores; WISC, Weschler intelligence scale for children.

#### Statistical techniques

Because the numbers of women consuming paracetamol daily were relatively small (Table 1) we have considered any consumption in each period as the exposures for initial analyses. Previously we have shown, using an exposome analysis, that 15 variables were independently associated with taking paracetamol in the period 18–32 weeks gestation. These included variables that were indications for use such as headache, influenza and back pain. Lifestyle factors shown to be independently associated with paracetamol intake included alcohol consumption and diet. Interestingly none of the traditional measures of social disadvantage were associated with paracetamol intake once the 15 factors had been taken into account; it is possible that the combined health and behaviour characteristics were themselves equivalent to an indicator of social disadvantage (Golding et al., 2020). The 15 factors were all used as potential confounders in our previous study of child behaviours (Golding et al., 2020). Here we use the same potential confounders and analyse the 23 outcomes related to educational attainment using multiple regression in STATA version 14. The results are presented with the regression coefficient (i.e., mean difference) with its 95% confidence interval (CI). It should be noted that the standardised regression coefficients of the adjusted data are also available in the Supplementary Material.

In this study, with its emphasis on identifying possible adverse effects of paracetamol on the developing child, we were anxious that results would not be ignored because of possible Type II or even Type I errors. Consequently, we have used both the number of results at *p* < 0.05 (thus avoiding Type II errors), and the *p*-value using the Bonferroni correction (to avoid Type I errors). Because we were testing for adverse effects, one-tailed tests were used in instances when the overall *p*-value was <0.10. In order to take account of multiple testing we referred to the Bonferroni correction calculated as *p* = 0.004. Sensitivity analyses used the E-value statistics calculated in STATA (Linden et al., 2019). Interpretation of the size of the E-values has used the relative sizes of the E-values within each table as suggested by VanderWeele and Mathur (2020). Thus, the larger the E-value the more unlikely that an unknown confounder would nullify the findings.

Since educational abilities are influenced by the age at which the child starts school (and therefore their month of birth), we tested whether the relationships between paracetamol exposures and months of birth were apparent in the adjusted analyses. No such associations were found and therefore month of birth was not taken into account. Since there is increasing evidence that associations and mechanisms vary between the sexes (e.g., Huang et al., 2023; Silveira et al., 2023), separate analyses were undertaken for each of the two sexes as defined at birth.

## Results

### Scholastic abilities

The numbers involved in the 23 unadjusted and adjusted analyses for each of the times of exposure are shown in Table 3. This shows that the range of numbers available for unadjusted and adjusted analysis were greatest for the national test results (KS2 and KS3) for maths and reading, and lowest for ALSPAC-devised reading and maths tests at ages 12 years and school Year 8 respectively. All adjusted analyses had n > 1,500 children.

**TABLE 3:** Numbers of pregnancies for which there is data on both maternal paracetamol intake and scholastic outcomes.

Scholastic measure	N^a^	N^b^	N^c^	N^d^
Reading score at 7	7,488	6,035	7,204	6,033
Reading score at 9	7,055	5,715	6,806	5,719
Comprehension score at 9	6,422	5,181	6,181	5,187
Reading speed at 9	6,405	5,171	6,169	5,177
Reading accuracy at 9	6,422	5,181	6,181	5,187
Reading speed at 12	1,868	1,528	1,832	1,529
Reading speed at 13	5,151	4,251	4,991	4,254
Phoneme score at 7	7,470	6,026	7,188	6,024
Non-word reading at 9	7,042	5,708	6,794	5,712
Non-word reading at 12	1,865	1,526	1,829	1,527
Non-word reading at 13	5,136	4,239	4,978	4,242
SATs reading assessment at 10-11	8,801	6,469	8,136	6,475
KS3 reading test at 13-14	9,568	7,115	8,881	7,116
KS3 Shakespeare reading at 13-14	9,449	7,043	8,772	7,043
Spelling score at 7	7,375	5,955	7,098	5,953
Spelling score at 9	7,040	5,705	6,789	5,709
WISC arithmetic score at 8	6,833	5,565	6,606	5,564
Year 4 maths test score at 8	4,796	3,646	4,449	3,647
Year 6 maths test score at 11	7,218	5,426	6,729	5,432
Year 8 maths test score at 12	2,545	1,968	2,398	1,975
KS2 maths test score at 10-11	10,998	8,515	10,226	8,222
KS3 maths test score at 13-14	9,726	7,218	9,025	7,218
Science reasoning score at 11-12	7,238	5,460	6,751	5,464

^a^ Numbers with paracetamol prior to 18 weeks and scholastic test outcomes.

^b^ Numbers with paracetamol <18 weeks, scholastic outcomes and confounders (mother ever having asthma, indigestion, back pain or migraine, pre-pregnancy BMI, subjective assessment of health in late pregnancy, having a cold, flu, an infection or a headache in late pregnancy, healthy diet score, processed diet score, alcohol consumption in pregnancy, domestic cleaning chemical score and parity).

^c^ Numbers with paracetamol between 18 and 32 weeks, and scholastic outcomes.

^d^ Numbers with paracetamol 18–32 weeks, scholastic outcomes and confounders (mother ever having asthma, indigestion, back pain or migraine, pre-pregnancy BMI, subjective assessment of health in late pregnancy, having a cold, flu, an infection or a headache in late pregnancy, healthy diet score, processed diet score, alcohol consumption in pregnancy, domestic cleaning chemical score and parity). KS2 = Key stage 2; KS3 = Key stage 3 (see Supplementary material); SATs, Standardised Attainment Test Scores; WISC, Weschler intelligence scale for children.

The results of unadjusted and adjusted analyses for the two time periods during which mothers took paracetamol are shown in Table 4 and Table 5. For exposure prior to 18 weeks, unadjusted associations with the 23 scholastic tests were all negative, and 20 of them showed *p* < 0.05. After adjustment, however, although 21 test results were negative, only five were at *p* < 0.05, compared with the 1.15 results expected. Interestingly these all involved tests at secondary school ages (11+) (Table 4). Among sex specific adjusted analyses for exposures <18 weeks (Table 5), there were three associations at *p* < 0.05 among the boys, and four among the girls; these numbers of associations were slightly more than the 1.15 expected (5% of 23) for each sex. There was no indication of any interaction between the sexes.

**TABLE 4:** Unadjusted and adjusted associations of paracetamol <18 weeks with measures of offspring scholastic ability; the mean differences indicate the difference in mean test score between the offspring exposed to paracetamol minus the score that would have been expected. A negative mean difference indicates that the exposed offspring had a lower score than expected.

Scholastic test	Unadjusted		Adjusted	
	Mean diff [95% CI]	*P*	Mean diff [95% CI]	*P*
Reading score at 7	−0.742 [-1.168, −0.317]	**0.001**	−0.287 [-0.758, 0.185]	0.234
Reading score at 9	−0.070 [-0.186, 0.046]	0.235	−0.004 [-0.132, 0.123]	0.949
Comprehension score at 9	−0.611 [-0.994, −0.227]	**0.002**	−0.097 [-0.510, 0.317]	0.647
Reading speed at 9	−2.059 [-3.419, −0.700]	**0.003**	−0.390 [-1.900, 1.120]	0.613
Reading accuracy at 9	−1.225 [-2.227, −0.223]	**0.017**	0.109 [-0.991, 1.209]	0.846
Reading speed at 12	−0.995 [-1.959, −0.031]	**0.043**	−0.522 [-1.642, 0.597]	0.360
Reading speed at 13	−0.716 [-1.282, −0.151]	**0.013**	−0.065 [-0.701, 0.572]	0.842
Phoneme score at 7	−0.155 [-0.589, 0.278]	0.482	−0.170 [-0.661, 0.322]	0.499
Non-word reading at 9	−0.050 [-0.167, 0.067]	0.402	0.020 [-0.113, 0.153]	0.769
Non-word reading at 12	−1.165 [-2.041, −0.290]	**0.009**	−0.982 [-1.996, 0.033]	**0.029** ^b^
Non-word reading at 13	−0.556 [-1.068, −0.044]	**0.033**	−0.009 [-0.585, 0.567]	0.976
SATs reading assessment at 10-11	−0.069 [-0.106, −0.032]	**<0.001**	−0.025 [-0.067, 0.018]	0.257
KS3 reading test at 13-14	−0.719 [-0.978, −0.461]	**<0.001**	−0.320 [-0.607, −0.032]	**0.015^b^**
KS3 Shakespeare reading at 13-14	−0.387 [-0.528, −0.245]	**<0.001**	−0.120 [-0.282, 0.042]	0.073^b^
Spelling score at 7	−0.314 [-0.517, −0.112]	**0.002**	−0.160 [-0.390, 0.070]	0.086^b^
Spelling score at 9	−0.205 [-0.367, −0.042]	**0.014**	−0.052 [-0.233, 0.129]	0.576
WISC arithmetic score at 8	−0.199 [-0.363, −0.036]	**0.017**	−0.061 [-0.246, 0.123]	0.514
Year 4 maths test score at 8	−0.259 [-0.436, −0.082]	**0.004**	−0.168 [-0.371, 0.034]	0.052^b^
Year 6 maths test score at 11	−0.823 [-1.149, −0.497]	**<0.001**	−0.484 [-0.859, −0.109]	**0.006^b^**
Year 8 maths test score at 12	−0.636 [-1.195, −0.077]	**0.026**	−0.271 [-0.907, 0.365]	0.404
KS2 maths test score at 10-11	−2.334 [-3.136, −1.532]	**<0.001**	−1.301 [-2.201, −0.400]	**0.003^b^ **
KS3 maths test score at 13-14	−1.362 [-2.237, −0.486]	**0.002**	−0.255 [-1.271, 0.761]	0.623
Science reasoning at 11-12	−0.269 [-0.388, −0.151]	**<0.001**	−0.168 [-0.305, −0.030]	**0.009^b^**

^a^ Adjusted for mother ever having had asthma, indigestion, back pain or migraine, pre-pregnancy BMI, subjective assessment of health in late pregnancy, having a cold, flu, an infection or a headache in late pregnancy, healthy diet score, processed diet score, alcohol consumption in pregnancy, domestic cleaning chemical score and parity. *p*-values <.05 are in bold, and those less than the Bonferroni correction are underlined.

^b^ one-tailed test. KS2 = Key stage 2; KS3 = Key stage 3 (see Supplementary material); SATs, Standardised Attainment Test Scores; WISC, Weschler intelligence scale for children.

**TABLE 5:** Adjusted associations of maternal intake of paracetamol 18 weeks with sex-specific measures of offspring scholastic abilities; the mean differences indicate the difference in mean test score between the offspring exposed to paracetamol minus the score that would have been expected. A negative mean difference indicates that the exposed offspring had a lower score than expected.

Measure of scholastic abilities	Boys		Girls	
	Mean diff [95% CI]	*P*	Mean diff [95% CI]	*p*
Reading score at 7	−0.524 [-1.209, 0.162]	0.067^b^	−0.048 [-0.682, 0.586]	0.883
Reading score at 9	−0.025 [-0.214, 0.163]	0.791	0.009 [-0.162, 0.180]	0.918
Comprehension score at 9	−0.180 [-0.800, 0.440]	0.569	−0.0004 [-0.548, 0.547]	0.999
Reading speed at 9	−1.307 [-3.510, 0.897]	0.245	0.569 [-1.500, 2.638]	0.590
Reading accuracy at 9	−0.236 [-1.893, 1.355]	0.745	0.491 [-0.995, 1.976]	0.517
Reading speed at 12	0.607 [-1.028, 2.241]	0.466	−1.915 [-3.441, −0.388]	**0.007^b^**
Reading speed at 13	0.145 [-0.794, 1.084]	0.762	−0.328 [-1.184, 0.529]	0.453
Phoneme score at 7	−0.225 [-0.921, 0.471]	0.527	−0.117 [-0.813, 0.578]	0.741
Non-word reading at 9	−0.0003 [-0.192, 0.191]	0.997	0.030 [-0.155, 0.214]	0.751
Non-word reading at 12	−0.432 [-1.880, 1.015]	0.558	−1.613 [-3.052, −0.173]	**0.014^b^**
Non-word reading at 13	0.064 [-0.780, 0.909]	0.881	−0.108 [-0.895, 0.679]	0.789
SATs reading assessment at 10-11	−0.037 [-0.097, 0.023]	0.230	−0.019 [-0.079, 0.041]	0.526
KS3 reading test at 12-13	−0.390 [-0.789, 0.009]	0.028^b^	−0.304 [-0.702, 0.094]	0.067^b^
KS3 Shakespeare reading at 12-13	−0.228 [-0.451, −0.004]	**0.023^b^**	−0.056 [-0.283, 0.170]	0.626
Spelling score at 7	−0.355 [-0.678, −0.031]	**0.016^b^**	0.058 [-0.261, 0.378]	0.720
Spelling score at 9	−0.125 [-0.400, 0.150]	0.374	−0.001 [-0.233, 0.231]	0.994
WISC arithmetic score at 8	−0.045 [-0.319, 0.229]	0.748	−0.063 [-0.310, 0.184]	0.617
Year 4 maths test score at 8	−0.059 [-0.347, 0.229]	0.687	−0.279 [-0.563, 0.006]	**0.028^b^**
Year 6 maths test score at 11	−0.390 [-0.921, 0.141]	0.075^b^	−0.490 [-1.013, 0.033]	0.039^b^
Year 8 maths test score at 12	−0.204 [-1.070, 0.663]	0.645	−0.231 [-1.162, 0.700]	0.627
KS2 maths test score at 10-11	−1.092 [-2.354, 0.169]	0.045^b^	−1.445 [-2.728, −0.162]	**0.019^b^**
KS3 maths test score at 13-14	−0.197 [-1.605, 1.210]	0.783	−0.188 [-1.656, 1.280]	0.802
Science reasoning at 11-12	−0.243 [-0.436, −0.050]	**0.007^b^**	−0.094 [-0.291, 0.102]	0.348

^a^ Adjusted for mother ever having asthma, indigestion, back pain or migraine, pre-pregnancy BMI, subjective assessment of health in late pregnancy, having a cold, flu, an infection or a headache in late pregnancy, healthy diet score, processed diet score, alcohol consumption in pregnancy, domestic cleaning chemical score and parity.

^b^ one-tailed test. *p*-values <.05 are in bold. KS2 = Key stage 2; KS3 = Key stage 3 (see Supplementary material); SATs, Standardised Attainment Test Scores; WISC, Weschler intelligence scale for children.

For exposures between 18 and 32 weeks (Table 6), all unadjusted associations with the scholastic tests were negative and all were at *p* < 0.05. After adjustment, three of the test results at age 9 became positive but the remaining 20 results remained negative; 12 of the 20 negative results were at *p* < 0.05, and six at *p* < 0.01. It is interesting that there were no associations at *p* < 0.05 with spelling ability or arithmetic. When sex-specific adjusted associations were compared (Table 7), none of the tests undertaken by the boys were at *p* < 0.05, whereas 12 of the associations for the girls were at *p* < 0.05, eight of these being at *p* < 0.01, and two at *p* < 0.001. These mostly involved tests at secondary school age. The adjusted tests that had *p* values less than the Bonferroni cut point (0.004) were all concerned with mathematics.

**TABLE 6:** Unadjusted and adjusted associations of paracetamol 18–32 weeks with measures of offspring cognition; the mean differences indicate the difference in mean test score between the offspring exposed to paracetamol minus the score that would have been expected. A negative mean difference indicates that the exposed offspring had a lower score than expected.

Measure of scholastic ability	Unadjusted		Adjusted	
	Mean diff [95% CI]	*P*	Mean diff [95% CI]	*p*
Reading score at 7	−1.213 [-1.648, −0.779]	**<0.001**	−0.542 [-1.047, −0.037]	**0.018^b^**
Reading score at 9	−0.187 [-0.306, −0.068]	**0.002**	−0.034 [-0.170, 0.103]	0.628
Comprehension score at 9	−0.756 [-1.151, −0.361]	**<0.001**	0.108 [-0.333, 0.549]	0.631
Reading speed at 9	−2.269 [-3.670, −0.868]	**0.002**	0.408 [-1.203, 2.018]	0.620
Reading accuracy at 9	−1.810 [-2.842, −0.778]	**0.001**	0.063 [-1.112, 1.238]	0.916
Reading speed at 12	−1.688 [-2.672, −0.704]	**0.001**	−1.034 [-2.232, 0.164]	**0.046^b^**
Reading speed at 13	−1.109 [-1.688, −0.529]	**<0.001**	−0.340 [-1.020, 0.339]	0.326
Phoneme score at 7	−0.721 [-1.166, −0.276]	**0.002**	−0.624 [-1.151, −0.097]	**0.010^b^**
Non-word reading at 9	−0.163 [-0.283, −0.042]	**0.008**	−0.008 [-0.150, 0.134]	0.911
Non-word reading at 12	−1.903 [-2.796, −1.010]	**<0.001**	−1.533 [-2.617, −0.449]	**0.003^b^ **
Non-word reading at 13	−0.815 [-1.342, −0.288]	**0.002**	−0.060 [-0.676, 0.556]	0.848
SATs reading assessment at 10-11	−0.114 [-0.153, −0.076]	**<0.001**	−0.054 [-0.099, −0.009]	**0.009^b^**
KS3 reading test at 13-14	−0.964 [-1.231, −0.697]	**<0.001**	−0.382 [-0.687, −0.077]	**0.007^b^**
KS3 Shakespeare reading at 13-14	−0.479 [-0.626, −0.333]	**<0.001**	−0.149 [-0.321, 0.023]	**0.045^b^**
Spelling score at 7	−0.419 [-0.626, −0.213]	**<0.001**	−0.124 [-0.371, 0.122]	0.323
Spelling score at 9	−0.270 [-0.437, −0.103]	**0.002**	−0.021 [-0.215, 0.173]	0.831
WISC arithmetic score at 8	−0.343 [-0.510, −0.176]	**<0.001**	−0.136 [-0.334, 0.062]	0.089^b^
Year 4 maths test score at 8	−0.364 [-0.548, −0.179]	**<0.001**	−0.213 [-0.429, 0.003]	**0.027^b^**
Year 6 maths test score at 11	−1.152 [-1.490, −0.814]	**<0.001**	−0.580 [-0.977, −0.183]	**0.002^b^ **
Year 8 maths test score at 12	−1.038 [-1.615, −0.462]	**<0.001**	−0.231 [-0.901, 0.439]	0.500
KS2 maths test score at 10-11	−3.292 [-4.116, −2.469]	**<0.001**	−1.626 [-2.577, −0.675]	**0.0005^b^ **
KS3 maths test score at 13-14	−2.752 [-3.660, −1.845]	**<0.001**	−1.378 [-2.454, −0.302]	**0.006^b^**
Science reasoning at 11-12	−0.315 [-0.439, −0.192]	**<0.001**	−0.132 [-0.278, 0.014]	**0.038^b^**

^a^ Adjusted for mother ever having had. asthma, indigestion, back pain or migraine, pre-pregnancy BMI, subjective assessment of health in late pregnancy, having a cold, flu, an infection or a headache in late pregnancy, healthy diet score, processed diet score, alcohol consumption in pregnancy, domestic cleaning chemical score and parity.

^b^ one-tailed test. *p*-values <.05 are in bold, and those less than the Bonferroni correction are underlined. KS2 = Key stage 2; KS3 = Key stage 3 (see Supplementary material); SATs, Standardised Attainment Test Scores; WISC, Weschler intelligence scale for children.

**TABLE 7:** Adjusted associations of maternal intake of paracetamol at 18–32 weeks with sex-specific measures of offspring scholastic abilities; the mean differences indicate the difference in mean test score between the offspring exposed to paracetamol minus the score that would have been expected. A negative mean difference indicates that the exposed offspring had a lower score than expected.

Measure of scholastic abilities	Boys		Girls	
	Mean diff [95% CI]	*P*	Mean diff [95% CI]	*p*
Reading score at 7	−0.291 [-1.037, 0.455]	0.444	−0.779 [-1.447, −0.111]	**0.011^b^**
Reading score at 9	−0.014 [-0.219, 0.192]	0.896	−0.051 [-0.231, 0.130]	0.581
Comprehension score at 9	0.517 [-0.156, 1.190]	0.066^b^	−0.260 [-0.837, 0.316]	0.376
Reading speed at 9	0.742 [-1.649, 3.133]	0.543	0.088 [-2.087, 2.264]	0.937
Reading accuracy at 9	0.951 [-0.814, 2.715]	0.291	−0.706 [-2.269, 0.858]	0.376
Reading speed at 12	−0.305 [-2.090, 1.481]	0.738	−1.936 [-3.525, −0.346]	**0.009^b^**
Reading speed at 13	−0.405 [-1.421, 0.610]	0.434	−0.331 [-1.235, 0.574]	0.473
Phoneme score at 7	−0.375 [-1.133, 0.382]	0.331	−0.880 [-1.613, −0.147]	**0.010^b^**
Non-word reading at 9	0.065 [-0.144, 0.273]	0.543	−0.084 [-0.278, 0.110]	0.398
Non-word reading at 12	−1.149 [-2.726, 0.429]	0.077^b^	−2.063 [-3.559, −0.566]	**0.004^b^ **
Non-word reading at 13	−0.015 [-0.928, 0.899]	0.975	−0.154 [-0.985, 0.677]	0.716
SATs reading assessment at 10-11	−0.040 [-0.104, 0.025]	0.226	−0.074 [-0.136, −0.012]	**0.010^b^**
KS3 reading test at 13-14	−0.318 [-0.747, 0.111]	0.073^b^	−0.514 [-0.930, −0.098]	**0.008^b^**
KS3 Shakespeare reading at 13-14	−0.076 [-0.316, 0.163]	0.532	−0.272 [-0.510, −0.034]	**0.013^b^**
Spelling score at 7	−0.091 [-0.444, 0.261]	0.612	−0.168 [-0.505, 0.169]	0.330
Spelling score at 9	0.124 [-0.175, 0.423]	0.417	−0.158 [-0.402, 0.086]	0.204
WISC arithmetic score at 8	−0.101 [-0.400, 0.199]	0.510	−0.158 [-0.418, 0.103]	0.235
Year 4 maths test score at 8	−0.021 [-0.329, 0.287]	0.895	−0.407 [-0.710, −0.105]	**0.004^b^ **
Year 6 maths test score at 11	−0.128 [-0.695, 0.440]	0.659	−0.983 [-1.531, −0.436]	**0.0002^b^ **
Year 8 maths test score at 12	−0.002 [-0.931, 0.926]	0.996	−0.366 [-1.330, 0.598]	0.456
KS2 maths test score at 10-11	−0.994 [-2.337, 0.350]	0.074^b^	−2.182 [-3.525, −0.839]	**0.0005^b^ **
KS3 maths test score at 13-14	−0.649 [-2.157, 0.860]	0.399	−2.007 [-3.543, −0.470]	**0.005^b^**
Science reasoning at 11-12	−0.060 [-0.267, 0.147]	0.570	−0.201 [-0.406, 0.005]	**0.028^b^**

^a^ Adjusted for mother ever having asthma, indigestion, back pain or migraine, pre-pregnancy BMI, subjective assessment of health in late pregnancy, having a cold, flu, an infection or a headache in late pregnancy, healthy diet score, processed diet score, alcohol consumption in pregnancy, domestic cleaning chemical score and parity.

^b^ one-tailed test. *p*-values <.05 are in bold, and those less than the Bonferroni correction are underlined. KS2 = Key stage 2; KS3 = Key stage 3 (see Supplementary material); SATs, Standardised Attainment Test Scores; WISC, Weschler intelligence scale for children.

In order to test the specificity of the results, the E-values are shown for the associations of Table 7 in Supplementary Material. In general, the E-values of the girls’ results were substantially higher for those who were shown to have adjusted associations at *p* < 0.05 (range 1.352–1.727) compared with those with *p* > 0.05 (range 1.057–1.267), implying that unknown confounders were less likely to have caused the associations at *p* < 0.05 than those at *p* > 0.05.

## Discussion

We have shown in this set of analyses that female (but not male) offspring of women who took paracetamol during the period from 18 to 32 weeks gestation were more likely to do less well on scholastic tests such as reading and mathematics. These results were robust and remained, particularly for mathematics, after adjustment for 15 potential confounders previously identified from an exposome analysis.

An earlier study, using this cohort, examined relationships between maternal paracetamol intake between 18- and 32-weeks gestation and 135 neurocognitive measures at ages from 6 months to 15 years. The measures mainly involved temperament, behaviour and cognition (Golding et al., 2020). We showed an increase in hyperactive behaviour and conduct disorders, in particular at around 3–4 years of age but not later; there were no differences between the sexes. In contrast the present study has shown associations with scholastic measures at later rather than earlier ages and involving girls rather than boys. Here we have also shown that paracetamol intake in the time period 18–32 weeks gestation was important for the future scholastic abilities of the child, and that this time window was more important for these outcomes than the first half of pregnancy. This suggests that the neuronal development during this fetal period provides an important structure for the child’s later learning abilities.

As with all observational studies, it is important that these scholastic results be confirmed in other longitudinal birth cohort studies. To our knowledge there have been no previous studies published which have addressed outcomes concerning scholastic competence. Assuming that there will be such evidence, either from other birth cohort studies, or from biological markers (e.g., genetic or epigenetic), there remains the question as to what the mechanism might be. Animal experiments have indicated that offspring of pregnant animals exposed to paracetamol had deficits in spatial learning and changed levels of BDNF (brain-derived neurotrophic factor) in specific parts of the neonatal brain (Viberg et al., 2014; McCrae et al., 2018). This may provide a clue as to a possible mechanism.

It may be considered that the effect sizes were too small to be worth considering, but it is worth noting that economic studies have shown that the loss of one IQ point (i.e. 0.067 standard deviations of the IQ distribution) has been estimated to result in a lifetime loss of earnings in the United States of $1,413,313 for boys and $1,156,157 for girls (Boyle et al., 2021). It is therefore possible that the loss of similar proportions of scores on reading and mathematics (which are key to many types of employment) may have an even more striking monetary effect.

However, one puzzle is why the association was found amongst girls but not boys. Previous studies have indicated that paracetamol intake in pregnancy is associated with hyperactivity, atopic and allergic disease, and disorders of sexual development such as early onset of puberty in humans (e.g., Bauer et al., 2021) and sexual behaviour in animal experiments (Holm et al., 2016; Kilcoyne and Mitchell, 2017). Could the sexual changes be a marker of gender-specific brain differences during fetal development that have resulted in girls exposed to paracetamol in pregnancy being less proficient in mathematics? Further follow-up of this cohort is required to assess whether the findings we have demonstrated at ages up to 14 years are continued until 16 and 18 years when the next national tests took place.

### Strengths and limitations

The strengths of this study lie in: a) the relatively large numbers of individuals tested which are representative of the population, especially for results based on the National tests (KS2 and KS3); b) the results used in this study are based on independent tests, marked objectively (i.e., not based on potentially biased views of parents or teachers); c) the analyses allow for the indications for paracetamol use as well as demographic variables, and were identified previously using an exposome analysis.

The limitations concern: a) that we were only able to look at two time periods of exposure, and were unable to assess the associations with exposures after 32 weeks gestation; b) that we did not have sufficient data to be able to determine whether there were dose effects; c) we were unable to compare our results of paracetamol intake with that of other analgesics since the frequency with which aspirin and other medications were used was considerably less than would have been appropriate to use as a control population (e.g., only 670 women were taking aspirin in the first half of pregnancy compared with 7,163 who took paracetamol); d) as with most observational studies, it is possible that there was residual confounding even though taking account of 15 different independent factors representing indications for use of the drug, lifestyle and biological features of the woman; as a sensitivity analysis we repeated the analyses including maternal education as a confounder, but this made no difference to the *p* values shown here. E-values were also computed although it is recognised that interpretation of such values when using continuous variables is problematic. Nevertheless, they are included in Supplementary material for the interested reader; e) that the population concerned was predominantly white European, and consequently the data cannot be extrapolated to the world-wide population. Nevertheless, it does provide a warning that wherever possible paracetamol should be avoided during pregnancy.

In 2021, Ann Bauer of the Department of Public Health, University of Massachusetts School of Health Sciences in the USA, reviewed the literature and published a consensus statement signed by 90 scientists (https://doi.org/10.1038/s41574-021-00553-7) recommending ‘that pregnant women should be cautioned at the beginning of pregnancy to: forego [paracetamol] unless its use is medically indicated; consult with a physician or pharmacist if they are uncertain whether use is indicated and before using on a long-term basis; and minimize exposure by using the lowest effective dose for the shortest possible time.’ A reply was subsequently published by Alwan et al. (2022) pouring scorn on the data available and stating that:

‘Until better data for systematic synthesis become available, we urge against recommending such precautionary measures for [paracetamol] use in pregnancy and against the dissemination of information based on inconclusive and insufficient evidence. At the same time, we strongly encourage that such questions related to the safety of paracetamol use in pregnancy should continue to be investigated.’ This received the response from Bauer et al. (2022): ‘We agree that limitations and uncertainties remain despite the large body of available data, therefore, we avoided any inference of causality in our Consensus Statement. We believe, however, that available data provide sufficient evidence for concern and a recommendation of precautionary action.’ They went on to say that ‘the large body of experimental data, mostly consistent with observational data, is an important consideration in our evaluation.’

It is in the light of these discussions that we have analysed the scholastic outcomes from the ALSPAC longitudinal data. As far as we are aware there have been no previous studies concerning abilities in reading, spelling, science or mathematics. Our results therefore need to be corroborated by other longitudinal studies. However, we concur with Bauer and colleagues that pregnant women should be warned against taking paracetamol during pregnancy, if possible to do so.

## Conclusion

We have demonstrated that paracetamol ingestion in the latter part of pregnancy is associated with poorer outcomes in scholastic achievement in reading and mathematics among girls of secondary school age. We conclude that mounting evidence suggests that use of this medication should be discouraged during pregnancy.

## Data Availability

The data analyzed in this study is subject to the following licenses/restrictions: Access to the data requires the permission of the ALSPAC Executive Committee. Requests to access these datasets should be directed to alspac-exec@bristol.ac.uk.
