# Supplementary material for: Maternal prenatal paracetamol ingestion and scholastic attainments of the offspring
Source: Front Pharmacol. 2023 Dec 12;14:1116683. doi: 10.3389/fphar.2023.1116683 (PMC10749925; doi:10.3389/fphar.2023.1116683)
Supplement: Supplementary file 1 [file DataSheet1.pdf]

Supplementary Material

Maternal prenatal paracetamol ingestion and scholastic attainments of the offspring

Jean Golding, Holly Tunstall, Steven Gregory, Yasmin Iles-Caven

Contents

I. The Outcome Measures
II. The Descriptive Tables of associations between possible confounders and paracetamol ingestion
III. Tables of adjusted standardised mean differences and E-values
IV. References

I. The Scholastic Abilities Outcome Measures

The descriptions below are taken verbatim from the paper by Golding et al (2020)

a) Spelling

"At both the 7- and the 9-year clinics, a spelling test was administered immediately after the reading session by trained ALSPAC staff. A total of 15 words were chosen specifically for each age group after piloting on several hundred children by Peter Bryant and Terezinha Nunes of the Department of Education at the University of Oxford. The spellings involved regular and irregular words of different frequencies. They were given in order of increasing difficulty. For each, the word was read out aloud to the child, and then within a specific sentence incorporating the word, and then alone again. The child was asked to write down the spelling even if he/she thought they were just guessing. The spelling score was the number of words spelt correctly (range 0-15). The scores for each age included those who stopped early (usually because they had reached the limit of their ability) (Nunes et al., 2012). The scores were not normally distributed but did not have ceiling effects."

b) Reading

Word reading at age 7 used the basic reading subtest of the WORD (Wechsler Objective Reading Dimension) for all children attending the ALSPAC clinic (Rust et al., 1993). The child was shown a series of four pictures by trained psychologists. Each picture had four short simple words underneath it. The child was asked to point to the word which had the same beginning or ending sound as the picture. This was then followed by a series of three pictures, each with four words beneath, each starting with the same letter. The child was asked to point to the word that correctly named the picture. Finally, the child was asked to read aloud a series of 48 unconnected words which increased in difficulty. If the child read the word incorrectly but pronounced it in a way that was phonetically plausible, this was also noted for each word. The reading task was stopped after the child had made six consecutive errors. 8070 children completed the task; the score was the number of items the child responded to correctly. It ranged from 0 to 54, with mean 28.1 (SD 9.44).

Reading words and non-words. At age 9 years, the child was asked to read aloud ten real words, followed by ten non-words. Both the words and non-words were selected from a larger selection of words taken from previous research (Nunes et al., 2003). The two sets of words were specifically

chosen for this study by Nunes and Bryant. The test–retest reliability of the word reading test is 0.8, and the scale has a correlation of 0.85 with the Schonell Word Reading Task (Schonell and Goodacre, 1971). Under test conditions, the child was shown each word in turn and asked to read the words aloud. The word reading score was calculated as the number of words read correctly. The score ranged from 0 to 10 and demonstrated a ceiling effect.

Reading comprehension was also measured at 9 years using the revised Neale Analysis of Reading Ability (NARA II) (Neale, 1997) to assess the child's reading skills and comprehension. It was administered by trained psychologists using Form II. The testing took place in a quiet room. Wherever possible, parents were asked not to accompany their child into the testing room to minimize distractions and interruptions. A similar structure was used for all test passages. A booklet was used from which each child read a passage. They were then asked a series of questions about the content of the story they had just read. The tester recorded the time (in seconds) it took the child to read the passage. Any errors made by the child during reading were noted on the data sheet. The child was prompted by the tester if they: (a) mispronounced a word; (b) substituted a word; (c) refused to say a word; (d) made an addition (only if it altered the meaning of the story); (e) made an omission; or (f) reversed a word. Administration of the test was undertaken following the instruction manual. If the child made more than 17 errors on the practice passage, the tester did not ask the child the comprehension questions but moved straight on to the level one story. All other children moved on to the level two story unless the tester felt that they had difficulty with reading the practice passage. If the child made less than three errors on the level two story the tester moved on to level three. If, however, the child made three or more errors on level two, the comprehension questions were administered but the tester moved down to the level one story (only moving on to level three if the child completed level one within the permissible number of errors). For the remaining test passages the child was not asked the comprehension questions if they made more than 16 errors (20 on level six) and the session was ended. The comprehension questions were asked as soon as the child had finished reading. For each question the child was given 10-12 seconds to respond; they could refer to the text to assist them. The raw comprehension score was obtained by summing the number of correct answers the child gave for each passage. If the permissible number of errors was exceeded for the final passage, the comprehension questions were not asked so no score was given for that passage. The conversion of the raw score to a score standardised for age used the test authors' criteria. It was approximately normally distributed. It should be noted that 48 children were unable to attempt the test and have been excluded.

Reading speed was calculated from the comprehension reading task. The rate of words per minute was computed for each child. This was based on only those passages read where no more than 16 errors were made (20 for passage 6) and was created as:

$$\textit{Total no. words read} \times 60 \,/\, \textit{Total time taken (sec)}$$

The reading speed standardised for age was approximately normally distributed with mean 105.1 (SD 12.6) words per minute.

Reading fluency or sight-word efficiency was estimated at the 13-year assessments. A word reading task (the TOWRE task) provides a test of sight-word efficiency (Torgesen et al., 1999). A list of 104 words was given to the child to read, and the number read accurately within 45 seconds was recorded. The score, identifying the number of words read in the time (but not necessarily the accuracy), was approximately normally distributed.

National tests of reading proficiency. The study linked to the offspring's national test results known as Key Stage 3. The 14-year-old assessments were administered at a mean age of 14 years 1 month. The reading tests comprised: (i) Reading a paper and answering questions; and (ii) reading a piece of Shakespeare and answering questions on its content.

c) Mathematics and Science

The development of numeracy and mathematical skills is made up of several components which are built on in hierarchical ways over time (Duckworth, 2008). Even before children enter formal schooling, they intuitively start to piece together basic mathematical concepts such as relative size and counting. Much of the research on the development of mathematical skills has focussed on arithmetic or word problem solving but little is known about influences on the general course of mathematics performance in non-selected populations. To obtain data appropriate to the aims of ALSPAC, various advisors were asked for their opinions over time. These included expert researchers including Terezhina Nunes and Peter Bryant, as well as representatives of the local Avon Education Authorities and other expert contributors. The result was the recommendation to use tests devised for the study by Nunes and Bryant which would ensure the measurement of mathematical reasoning.

Mathematical reasoning. The aim of these tasks was to assess children's understanding and use of quantitative relations to solve mathematical problems. There were two different Mathematical Reasoning tasks: one, containing 17 items, was given to schoolchildren in Year 4 (mean age 8 years 9 months); the other, containing 35 items, was given to children in Year 6 (mean age 11 years 2 months) and again in Year 8 (mean age 12 years 8 months). The aim of these tasks was to assess children's reasoning about quantities and the relations between quantities in mathematical problems independently of their computational skills. None of the items in these tests contained difficult calculations, the children had to reflect on the relations between quantities in each item to decide how to solve the problem. All the items were presented with the support of drawings; the children could use counting to solve many of the problems if they did not know the number facts that might be used in the solution. All the problems were presented orally by the teachers to avoid an undue influence of reading difficulties on the children's performance (Nunes et al., 2009).

Three types of item were included in the Year 4 Mathematics Reasoning Task: additive reasoning items about quantities; additive reasoning items about relations; and multiplicative reasoning items about quantities. The assessments used in Years 6 and 8 included six types of item: additive reasoning items about quantities; additive reasoning items about relations; multiplicative reasoning items about quantities; multiplicative reasoning items involving relations (i.e. proportions); items about spatial reasoning and items about fractional quantities. Analyses of their internal consistency using Cronbach's α showed that on all three occasions the mathematics reasoning tasks had good levels of inter-item reliability: 0.74 at Year 4 (N=5275), 0.89 at Year 6 (N=7881) and 0.91 at Year 8 (N=2755). This high internal consistency justifies the addition of all the items in each school year into single scores.

Arithmetical ability was measured as part of the WISC verbal intelligence tests at age 8 years. The raw scores at age 8 were measured using alternate questions as for the WISC test in general (Golding et al., 2017). The data were approximately normally distributed.

National tests of mathematics. The results of the school-administered Standardised Attainment Test Scores (SATS) at age 10-11 years (known as "key stage 2", KS2) and at 13-14 years (known as "key stage 3", KS3) were linked to the relevant children. These tests were scored in each subject at levels 1-8. The UK government recommendations are that children should achieve a level 4 by KS2 and a level 5 by KS3 and that they should progress by at least 1.0 unit or level every 2 years (National curriculum assessments, 2007). Thus, a coefficient of 0.5 represents an average 1 year of progress.

Scientific reasoning. A test of scientific reasoning was developed specifically for the study by Nunes and Bryant. The aim was to measure children's understanding that in a properly controlled scientific comparison, one variable is tested at a time while other variables are held constant. The test was administered in school Year 6 (age 11-12 years) by the class teacher. The pupils' scores successfully predicted their later progress in science even after allowance was made for age and IQ (Bryant et al., 2015)."

II.Descriptive Tables

For the studies on paracetamol intake during pregnancy, we started by using an exposome scan to identify those variables relating to the pregnant mothers which differed between those who did and did not take paracetamol between 18- and 32-weeks gestation at P<0.0001. We then dropped variables that did not contribute to a model predicting taking paracetamol and reduced the numbers of variables to 15 (described in Golding et al 2020). The tables below describe the differences between the women who took paracetamol and those who did not. Table S1 describes the various health conditions that they had, Tables S2 and S3 document the continuous and binary variables used to describe different facets of the women's lifestyles and social environments. The 15 factors that were included as confounders in the companion paper are in these tables together with others that are of interest. Those used as confounders are printed in red.

Table S1. The proportions of women having specific health conditions according to whether or not they took paracetamol in the period 18-32 weeks gestation. Independent factors used as confounders are printed in red.

**Health Condition**	**Took Paracetamol**		**OR [95%CI]**	**P**	**N**	**ALSPAC Variable**
**History of:**	**Yes**	**No**				
Hay fever	34.0%	29.5%	1.23 [1.13,1.23]	5.3e-7	11163	d150
Asthma	13.6%	9.9%	1.44 [1.29,1.62]	5.9e-10	11308	d153
Eczema	25.0%	21.9%	1.19 [1.09, 1.30]	1.0e-4	11257	d154
Indigestion	75.7%	66.2%	1.59 [1.47, 1.73]	1.1e-27	11186	d151
Back pain	52.6%	42.7%	1.47 [1.37,1.59]	2.9e-24	11324	d158
PID	3.1%	1.9%	0.62 [0.49, 0.79]	9.9e-5	11440	d166a
Hypertension	16.6%	12.7%	0.72 [0.65, 0.80]	1.6e-9	11217	d046
Migraine	53.7%	36.5%	2.01 [1.86, 2.16]	7.0e-72	11205	d157
**Had during 18-32 weeks**						
Poor health	32.8%	18.8%	1.59 [1.52, 1.67]	4.2e-94	11972	C050
A cold	48.5%	34.3%	1.79 [1.67, 1.93]	3.0e-54	11994	C058
Influenza	8.7%	3.8%	2.41 [2.06, 2.82]	7.0e-28	11994	C059
Any infection	32.3%	21.6%	1.74 [1.60,1.89]	8.1e-40	11994	C064
Headache	80.6%	46.4%	4.73 [4.36, 5.14]	5.7e-293	11994	C073
Back ache	85.2%	76.5%	1.74 [1.58,1.91]	2.3e-30	11994	C074
**Mental health Score at 32wk**	**Mean (SD)**	**Mean (SD)**	**Mean difference [95%CI]**	**P**	**N**	**ALSPAC variable**
Anxiety[a]	5.65	4.74 (3.45)	0.91 [0.78,1.04]	4.4e-40	11853	C574
Depression[b]	5.44	4.70 (3.13)	0.74 [0.63,0.86]	3.2e-43	11971	C601
Malaise[a]	16.72	14.23 (7.93)	2.49 [2.19,2.78]	1.6e-56	11850	C583

PID = Pelvic Inflammatory Disease; [a]Crown Crisp Experiential Index (Birtchnell et al., 1988); [b]EPDS (Cox et al., 1987).

Table S2. The mean measurements of environmental conditions of women according to whether or not they took paracetamol in the period 18-32 weeks gestation. Independent factors used as confounders are printed in red.

**Measurement**	**Took Paracetamol** Yes Mean (SD)	No Mean (SD)	**Mean difference [95%CI]**	**P**	**N**	**ALSPAC variable**
Prepregnant BMI	23.29 (4.09)	22.64 (3.55)	1.05 [1.04,1.06]	4.7e-18	10619	Dw042
Age at 1st pregnancy	24.04 (4.91)	24.98 (4.93)	0.96 [-.95, 0.97]	3.7e-24	11748	B023
Healthy diet score[a]	-.11 (.99)	.09 (1.00)	-.20 [-.23, -.16]	2.2e-28	11962	C00fac1
Processed diet score[a]	.07 (1.02)	-.05 (.98)	.12 [.09, .16]	4.7e-11	11962	C00fac3
No. cigarettes/day	.44 (.86)	.29 (.69)	1.28 [1.22,1.34]	3.9e-25	12001	C483
Domestic cleaning score[b]	11.44 (4.78)	10.53 (4.65)	.91 [.74, 1.09]	2.5e-24	11775	Achem
Household crowding (persons/room)	2.00 (.93)	1.79 (.90)	1.26 [1.21, 1.31]	5.0e-29	11459	A551
**Categorical social variables**						
Education level				2.5e-24	11931	C645a
Lowest	22.5	18.0	1.80 [1.58, 2.05]			
Vocational	9.9	9.8	1,47 [1.26, 1.71]			
O-level	34.7	34.8	1.44 [1.27, 1.62]			
A-level	22.5	22.5	1.44 [1.27,1.64]			
Highest	10.4	14.9	1.00 Ref			
Social class				0.041	10845	C765
I	2.9	3.9	1.00 Ref			
II	27.3	30.1	1.22 [.97, 1.53]			
IIINm	42.3	43.0	1.32 [1.06,1.65]			
IIIM	4.5	3.6	1.66 [1.24,2.21]			
IV	18.5	16.1	1.55 [1.22, 1.96]			
V	4.4	3.3	1.80 [1.34, 2.41]			

[a] For details of dietary scores see Northstone et al., 2008; [b] For details of domestic cleaning score see Sherriff et al., 2002.

Table S3. The proportion of women exposed to differing environmental conditions according to whether or not they took paracetamol in the period 18-32 weeks gestation. Independent factors used as confounders are printed in red.

**Exposures**	**Took Paracetamol Yes**	**No**	**OR [95%CI]**	**P**	**N**	**ALSPAC Variable**
Ever smoked regularly	53.6%	46.7%	1.31 [1.22, 1.41]	3.8e-13	11685	B650
Did not drink alcohol at 18 weeks	45.0%	53.7%	0.71[0.66, 0.76]	3.2e-20	11447	B722
Exposed to ETS[a]	65.1%	58.7%	1.32 [1.22, 1.43]	7.8e-12	10404	C481a
Lived in public housing	15.9%	10.3%	1.64 [1.47, 1.83]	5.3e-19	11625	A006
Worked during pregnancy	64.5%	72.5%	0.69 [0.64, 0.75]	8.7e-20	11347	C491
Parity 1+[b]	62.6%	49.4%	1.30 [1.25, 1.35]	1.4e-38	11560	B032

[a]ETS = environmental tobacco smoke or passive smoking; [b] Measures other children have been born to the mother prior to the current pregnancy.

III. Tables of adjusted standardised mean differences and E-values

Table S4. Standardised mean differences and e-values relating to the adjusted results for maternal paracetamol ingestion < 18 weeks gestation calculated from Table 4 of the main article

**Scholastic test**	**Adjusted Standardised mean diff [95% CI]**	**e-values PE, CI**
Reading score at 7	-0.030 [-0.080, 0.020]	1.198, 1.000
Reading score at 9	-0.002 [-0.053, 0.049]	1.041, 1.000
Comprehension at 9	-0.012 [-0.065, 0.096]	1.118, 1.000
Reading speed at 9	-0.014 [-0.069, 0.040]	1.127, 1.000
Reading accuracy at 9	0.005 [-0.048, 0.059]	1.074, 1.000
Reading speed at 12	-0.049 [-0.155, 0.056]	1.265, 1.000
Reading speed at 13	-0.006 [-0.068, 0.055]	1.081, 1.000
Phoneme score at 7	-0.018 [-0.069, 0.034]	1.145, 1.000
Non-word reading at 9	0.008 [-0.045, 0.061]	1.093, 1.000
Non-word reading at 12	-0.102 [-0.207, 0.003]	1.424, 1.000
Non-word reading at 13	-0.001 [-0.062, 0.060]	1.030, 1.000
SAT reading assessment	-0.028 [-0.076, 0.020]	1.188, 1.000
KS3 reading test	-0.049 [-0.094, -0.005]	1.265, 1.072
KS3 Shakespeare reading	-0.034 [-0.080, 0.012]	1.212, 1.000
Spelling score at 7	-0.036 [-0.088, 0.016]	1.219, 1.000
Spelling score at 9	-0.015 [-0.067, 0.037]	1.131, 1.000
WISC arithmetic at 8	-0.018 [-0.071, 0.036]	1.145, 1.000
Year 4 maths test score	-0.053 [-0.117, 0.011]	1.278, 1.000
Year 6 maths test score	-0.068 [-0.121, -0.015]	1.325, 1.134
Year 8 maths test score	-0.037 [-0.123, 0.050]	1.222, 1.000
KS2 maths test score	-0.060 [-0.102, -0.018]	1.300, 1.149
KS3 maths test score	-0.011 [-0.057, 0.034]	1.113, 1.000
Science reasoning at 11-12	-0.065 [-0.118, -0.012]	1.315, 1.116

PE = Point Estimate; CI = confidence Interval

Table S5. Standardised mean differences and e-values relating to the sex specific adjusted results for maternal paracetamol ingestion < 18 weeks gestation calculated from Table 5 of the main article.

**Measure of scholastic abilities**	**Boys**		**Girls**	
	**Standardised mean difference**	**e-values PE, CI**	**Standardised mean diff**	**e-values PE, CI**
Reading score at 7	-0.056 [-0.128, 0.017]	1.285, 1.000	-0.005 [-0.072, 0.062]	1.073, 1.000
Reading score at 9	-0.010 [-0.086, 0.065]	1.106, 1.000	0.004 [-0.065, 0.072]	1.061, 1.000
Comprehension score at 9	-0.023 [-0.102, 0.056]	1.168, 1.000	-0.00005 [-0.070, 0.070]	1.007, 1.000
Reading speed at 9	-0.047 [-0.127, 0.032]	1.258, 1.000	0.021 [-0.054, 0.095]	1.157, 1.000
Reading accuracy at 9	-0.013 [-0.092, 0.066]	1.122, 1.000	0.024 [-0.048, 0.096]	1.172, 1.000
Reading speed at 12	0.057 [-0.097, 0.211]	1.297, 1.000	-0.180 [-0.324, -0.037]	1.637, 1.223
Reading speed at 13	0.014 [-0.077, 0.105]	1.127, 1.000	-0.032 [-0.114, 0.051]	1.203, 1.000
Phoneme score at 7	-0.024 [-0.096, 0.049]	1.170, 1.000	-0.012 [-0.085, 0.060]	1.118, 1.000
Non-word reading at 9	-0.0001 [-0.077, 0.076]	1.011, 1.000	0.012 [-0.062, 0.086]	1.116, 1.000
Non-word reading at 12	-0.045 [-0.195, 0.105]	1.250, 1.000	-0.167 [-0.317, -0.018]	1.602, 1.148
Non-word reading at 13	0.007 [-0.083, 0.097]	1.086, 1.000	-0.011 [-0.095, 0.072]	1.113, 1.000
SAT reading assessment	-0.041 [-0.109, 0.026]	1.237, 1.000	-0.022 [-0.089, 0.045]	1.163, 1.000
KS3 reading test	-0.060 [-0.122, 0.001]	1.300, 1.000	-0.047 [-0.108, 0.014]	1.257, 1.000
KS3 Shakespeare reading	-0.065 [-0.128, -0.001]	1.314, 1.036	-0.016 [-0.080, 0.048]	1.137, 1.000
Spelling score at 7	-0.080 [-0.153, -0.007]	1.360, 1.088	0.013 [-0.059, 0.085]	1.122, 1.000
Spelling score at 9	-0.036 [-0.114, 0.043]	1.218, 1.000	-0.0002 [-0.067, 0.066]	1.015, 1.000
Arithmetic score at 8	-0.013 [-0.092, 0.066]	1.122, 1.000	-0.018 [-0.090, 0.053]	1.147, 1.000
Year 4 maths test score	-0.019 [-0.110, 0.072]	1.149, 1.000	-0.088 [-0.179, 0.002]	1.385, 1.000
Year 6 maths test score	-0.055 [-0.130, 0.020]	1.284, 1.000	-0.069 [-0.143, 0.005]	1.328, 1.000
Year 8 maths test score	-0.028 [-0.145, 0.090]	1.187, 1.000	-0.031 [-0.158, 0.095]	1.202, 1.000
KS2 maths test score	-0.050 [-0.109, 0.008]	1.269, 1.000	-0.067 [-0.126, -0.007]	1.321, 1.091
KS3 maths test score	-0.009 [-0.072, 0.054]	1.098, 1.000	-0.008 [-0.074, 0.057]	1.096, 1.000
Science reasoning at 11-12	-0.094 [-0.169, -0.019]	1.401, 1.153	-0.036 [-0.112, 0.040]	1.220, 1.000

PE = Point Estimate; CI=Confidence Interval

Table S6. Standardised mean differences and e-values relating to the adjusted results for maternal paracetamol ingestion 18 -32 weeks gestation calculated from Table 6 of the main article.

**Scholastic test**	**Adjusted**	
	**Standardised mean diff [95% CI]**	**e-values PE, CI**
Reading score at 7	-0.057 [-0.111, -0.004]	1.291, 1.064
Reading score at 9	-0.014 [-0.068, 0.041]	1.124, 1.000
Comprehension at 9	0.014 [-0.042, 0.070]	1.126, 1.000
Reading speed at 9	0.015 [-0.043, 0.073]	1.130, 1.000
Reading accuracy at 9	0.003 [-0.054, 0.060]	1.056, 1.000
Reading speed at 12	-0.097 [-0.210, 0.015]	1.411, 1.000
Reading speed at 13	-0.033 [-0.098, 0.033]	1.207, 1.000
Phoneme score at 7	-0.065 [-0.120, -0.010]	1.316, 1.107
Non-word reading at 9	-0.003 [-0.060, 0.053]	1.057, 1.000
Non-word reading at 12	-0.159 [-0.272, -0.047]	1.580, 1.257
Non-word reading at 13	-0.006 [-0.072, 0.059]	1.082, 1.000
SAT reading assessment	-0.061 [-0.112, -0.010]	1.303, 1.109
KS3 reading test	-0.059 [-0.106, -0.012]	1.296, 1.116
KS3 Shakespeare reading	-0.042 [-0.091, 0.006]	1.241, 1.000
Spelling score at 7	-0.028 [-0.083, 0.028]	1.188, 1.000
Spelling score at 9	-0.006 [-0.061, 0.049]	1.080, 1.000
Arithmetic score at 8	-0.039 [-0.097, 0.018]	1.231, 1.000
Year 4 maths test score	-0.067 [-0.136, 0.001]	1.323, 1.000
Year 6 maths test score	-0.082 [-0.138, -0.026]	1.366, 1.180
Year 8 maths test score	-0.031 [-0.122, 0.060]	1.202, 1.000
KS2 maths test score	-0.075 [-0.119, -0.031]	1.346, 1.201
KS3 maths test score	-0.062 [-0.110, -0.014]	1.305, 1.125
Science reasoning at 11-12	-0.051 [-0.107, 0.005]	1.271, 1.000

PE = Point Estimate; CI = Confidence Interval

Table S7. Standardised sex specific mean differences and e-values relating to the adjusted results for maternal paracetamol ingestion 18 -32 weeks gestation calculated from Table 7 of the main article.

**Measure of scholastic abilities**	**Boys**		**Girls**	
	**Standardised mean diff**	**e-values PE, CI**	**Standardised mean diff**	**e-values PE, CI**
Reading score at 7	-0.031 [-0.110, 0.048]	1.200, 1.000	-0.083 [-0.153, -0.012]	1.368, 1.116
Reading score at 9	-0.005 [-0.088, 0.077]	1.076, 1.000	-0.020 [-0.093, 0.052]	1.157, 1.000
Comprehension at 9	0.066 [-0.020, 0.152]	1.318, 1.000	-0.033 [-0.107, 0.040]	1.208, 1.000
Reading speed at 9	0.027 [-0.060, 0.113]	1.184, 1.000	0.003 [-0.075, 0.082]	1.057, 1.000
Reading accuracy at 9	0.046 [-0.040, 0.132]	1.255, 1.000	-0.034 [-0.110, 0.042]	1.213, 1.000
Reading speed at 12	-0.029 [-0.197, 0.140]	1.191, 1.000	-0.182 [-0.332, -0.033]	1.642, 1.208
Reading speed at 13	-0.039 [-0.137, 0.059]	1.230, 1.000	-0.032 [-0.119, 0.055]	1.204, 1.000
Phoneme score at 7	-0.039 [-0.119, 0.040]	1.231, 1.000	-0.092 [-0.169, -0.015]	1.396, 1.134
Non-word reading at 9	0.026 [-0.057, 0.109]	1.180, 1.000	-0.033 [-0.111, 0.044]	1.209, 1.000
Non-word reading at 12	-0.119 [-0.283, 0.044]	1.472, 1.000	-0.214 [-0.370, -0.059]	1.727, 1.298
Non-word reading at 13	-0.002 [-0.099, 0.095]	1.039, 1.000	-0.016 [-0.105, 0.072]	1.139, 1.000
SAT reading assessment	-0.044 [-0.116, 0.028]	1.249, 1.000	-0.083 [-0.153, -0.013]	1.370, 1.125
KS3 reading test	-0.049 [-0.115, 0.017]	1.264, 1.000	-0.079 [-0.143, -0.015]	1.358, 1.133
KS3 Shakespeare reading	-0.022 [-0.090, 0.046]	1.163, 1.000	-0.077 [-0.145, -0.010]	1.352, 1.104
Spelling score at 7	-0.021 [-0.100, 0.059]	1.157, 1.000	-0.038 [-0.114, 0.038]	1.225, 1.000
Spelling score at 9	0.035 [-0.050, 0.121]	1.217, 1.000	-0.045 [-0.115, 0.025]	1.251, 1.000
Arithmetic score at 8	-0.029 [-0.116, 0.058]	1.193, 1.000	-0.046 [-0.121, 0.030]	1.253, 1.000
Year 4 maths test score	-0.007 [-0.104, 0.091]	1.084, 1.000	-0.129 [-0.225, -0.033]	1.499, 1.210
Year 6 maths test score	-0.018 [-0.098, 0.062]	1.146, 1.000	-0.139 [-0.216, -0.061]	1.525, 1.305
Year 8 maths test score	-0.003 [-0.127, 0.126]	1.017, 1.000	-0.050 [-0.181, 0.081]	1.267, 1.000
KS2 maths test score	-0.046 [-0.108, 0.016]	1.254, 1.000	-0.101 [-0.163, -0.039]	1.421, 1.229
KS3 maths test score	-0.029 [-0.097, 0.039]	1.193, 1.000	-0.090 [-0.159, -0.021]	1.389, 1.160
Science reasoning at 11-12	-0.023 [-0.103, 0.057]	1.169, 1.000	-0.078 [-0.157, 0.002]	1.353, 1.000

PE = Point Estimate; CI = Confidence Interval

IV. References

Birtchnell, J, Evans, C, Kennard, J. (1988)The total score of the Crown-Crisp Experiential Index: a useful and valid measure of psychoneurotic pathology. *Br J Med Psychol.* 61: 255–66.

Bryant, P., Nunes, T., Hillier, J., Gilroy, C. and Barros, R. (2015). The importance of being able to deal with variables in learning science. *Int. J. Sci. Math. Educ*. 13(1), 145-163. https://doi.org/10.1007/s10763-013-9469-x

Cox, J. L., Holden, J. M., & Sagovsky, R. (1987). Detection of postnatal depression: development of the 10-item Edinburgh Postnatal Depression Scale. *The British journal of psychiatry*, *150*(6), 782-786.

Duckworth, K., 2008. The influence of context on attainment in primary school: Interactions between children, family and school contexts [Wider Benefits of Learning Research Report No. 28]. Centre for Research on the Wider Benefits of Learning, Institute of Education, University of London. https://discovery.ucl.ac.uk/id/eprint/10005964

Golding, J., Hibbeln, J.R., Gregory, S.M., Iles-Caven, Y., Emond, A. and Taylor, C.M., (2017). Maternal prenatal blood mercury is not adversely associated with offspring IQ at 8 years provided the mother eats fish: a British prebirth cohort study. *Int. J. Hygiene Environ. Health.* 220(7), 1161-1167. https://doi.org/10.1016/j.ijheh.2017.07.004

Golding, J., Gregory, S., Clark, R., Ellis, G., Iles-Caven, Y., & Northstone, K. (2020). Associations between paracetamol (acetaminophen) intake between 18 and 32 weeks gestation and neurocognitive outcomes in the child: A longitudinal cohort study. *Paediatric and Perinatal Epidemiology*, *34*(3), 257-266.

Golding, J., Gregory, S., Clark, R., Iles-Caven, Y., Ellis, G., Taylor, C.M. and Hibbeln, J. (2021). Maternal prenatal vitamin B12 intake is associated with speech development and mathematical abilities in childhood. *Nutr. Res*. 86, 68-78.

National curriculum assessments: Code of practice 2007. https://dera.ioe.ac.uk

Neale, M.D. (1997). Neale analysis of reading ability–revised. Windsor: NFER-Nelson.

Northstone, K., Emmett, P., & Rogers, I. (2008). Dietary patterns in pregnancy and associations with socio-demographic and lifestyle factors. *European journal of clinical nutrition*, *62*(4), 471-479.

Nunes, T., Bryant, P. and Olsson, J. (2003). Learning morphological and phonological spelling rules: An intervention study. *Sci. Stud. Read*. 7(3), 289-307. https://doi.org/10.1207/S1532799XSSR0703_6

Nunes, T., Bryant, P., Sylva, K. and Barros, R. (2009). Development of maths capabilities and confidence in primary school (No. Research Report DCSF-RR118). London: Department for Children, Schools and Families. ISBN 978 1 84775 457 8

Nunes, T., Bryant, P. and Barros, R. (2012). The development of word recognition and its significance for comprehension and fluency. J. *Educ. Psychol.* 104(4), 959. https://psycnet.apa.org/doi/10.1037/a0027412

Rust, J., Golombok, S. and Trickey, G. (1993). WORD: Wechsler objective reading dimensions manual. Sidcup, UK: Psychological Corporation. 074910290X (ISBN13: 9780749102906)

Schonell, F. and Goodacre, E. (1971). The psychology and teaching of reading. London: Oliver & Boyd. ISBN 978-0050026120

Sherriff, A., Golding, J., & Alspac Study Team. (2002). Factors associated with different hygiene practices in the homes of 15 month old infants. *Archives of disease in childhood*, *87*(1), 30-35.

Torgesen, J.K., Wagner, R. and Rashotte, C. (1999). TOWRE–2 Test of Word Reading Efficiency. Austin, TX: Pro-Ed.
